# Supplementary material for: Levels of complement factor H-related 4 protein do not influence susceptibility to age-related macular degeneration or its course of progression
Source: Nat Commun. 2024 Jan 10;15:443. doi: 10.1038/s41467-023-44605-0 (PMC10781981; doi:10.1038/s41467-023-44605-0)
Supplement: Supplementary file 1 — Supplementary Information [file 41467_2023_44605_MOESM1_ESM.pdf]

Content

Supplementary Table 1 Characteristics of the Utah & Iowa and IAMDGC case/control cohorts.

Supplementary Table 2 Frequencies of Chr1 (*CFH-CFHR5*) and Chr10 (*ARMS2/HTRA1*) variants associated with AMD among individuals with European ancestry from the 1000 Genomes Project phase 3 (1000 G EUR), Utah & Iowa and IAMDGC controls and cases, with associated effect sizes and p-values. Odds ratios, 95% confidence intervals (CI) and *p*-values (two-sided) were determined using logistic regressions using AMD case/control status as the dependent variable and age, sex and the first two genetic principal components for the IAMDGC cohort as covariates.

Supplementary Table 3 Independent *cis*-expression quantitative trait loci (*cis*-eQTL) for *CFHR4* transcript levels. These variants were identified by performing a conditional association analysis using genotype and liver gene expression levels for 183 donors (129 males, 54 females) with European ancestry (median age 56 IQR 14.5) from the Genotype-Tissue Expression Project (GTEx) dataset, version 8[1]. Minor allele frequencies and linkage disequilibrium statistics were determined among these GTEx liver donors. Only common variants (frequency > 5%) were considered. Conditioned effect sizes and *p*-values (two-sided) were obtained using a backward stepwise linear regression model. Effect size and strength of association with AMD were determined by using the IAMDGC case/control cohort (13,378 controls, 17,541 cases) while controlling for age, sex and the first two genetic principal components.

Supplementary Table 4 Independent *cis*-protein quantitative trait loci (*cis*-pQTL) identified for FHR-4. The study by Pietzner et al. (2021)[2] included 4,775 protein targets measured in plasma from 10,708 individuals with European ancestry (mean age 48.6 years, 53.3% women) from the Finland study. Association analyses were adjusted for age, sex, the first ten genetic principal components and test site. The study by (Gudjonsson et al. 2022)[3] included 4,782 protein targets measured in plasma collected from 5,368 individuals with European ancestry. Association analyses relied on linear regressions adjusted for age, sex, five genetic principal components and genotyping platforms. Effect size and strength of association with AMD were determined through logistic regression using the IAMDGC case/control

cohort (13,378 controls, 17,541 cases) while controlling for age, sex and the first two genetic principal components. The minor allele for each variant, which correspond to the alternative allele used to determine odds ratios (OR), 95% confidence intervals and *p*-value for association with AMD, is underlined. All *p*-values are two-sided.

Supplementary Table 5 Demographics of Utah & Iowa subjects among which FHR-4 plasma levels were measured, by Chr1 diplotype group and genotype at rs61818956, rs10494745 and rs7531555.

Supplementary Table 6 Demographics of Utah & Iowa eye donors used in this study, by genotype at rs61818956, rs10494745 and rs7531555.

Supplementary Table 7 Variants associated with systemic FHR-4 levels by GWAS and GWAS meta-analyses performed by Cipriani et al. (2020)[4], Cipriani et al. (2021)[5] and Lorés-Motta et al. (2021)[6] and independent sentinel *CFHR4* QTLs explaining these associations. The GWAS meta-analysis performed by Cipriani et al. (2020)[4] relied on 522 controls and 484 cases from the Cambridge & European Genetic Database (EUGENDA) cohorts. The GWAS on FHR-4 levels performed by Cipriani et al. (2021)[5] included 252 controls from the Cambridge cohort. The GWAS on FHR-4 levels carried out by Lorés-Motta et al. (2021)[6] relied on 202 controls from the EUGENDA cohort.

Supplementary Table 8 Comparison with studies from Cipriani et al. (2020)[4], Cipriani et al. (2021)[5] and Lorés-Motta et al. (2021)[6].

Supplementary Figure 1 Association between haplotypes based on rs800292 (*CFH* I62V), rs1410996 (IAMDGC Locus 1.1), rs1061170 (*CFH* Y402H, IAMDGC Locus 1.2) and the *CFHR3/1* deletion-tagging SNP rs12144939 (rs6677604 was used as a proxy for the IAMDGC cohort), with associated effect sizes and *p*-values. Frequencies among Europeans from the 1000 Genomes Project phase 3 (1000 G EUR) are also provided. AMD odds ratios (OR), 95% confidence intervals (CI) and *p*-values (two sided) were obtained by using logistic regressions including AMD case/control status as the dependent variable and for age, sex and the first two genetic principal components for the IAMDGC cohort as covariates. Score statistics

and *p*-values (two-sided) generated through chi-squared analyses are also shown. The IAMDGC Locus 1.1 imperfectly tags two independent protective haplotypes carrying the protective A allele at rs800292 or the deletion of *CFHR3/1*. On the protective haplotype H5, between 1.6% (IAMDGC cohort) and 1.9% (Utah & Iowa cohort) of controls do not carry the minor allele at the IAMDGC Locus 1.1 despite carrying the protective allele at rs800292. Bonferroni correction for multiple testing of 7 haplotypes = 0.0071 (0.05/7).

Supplementary Figure 2 Manhattan plot for single-variant associations with AMD conditioned on rs800292 (*CFH* I62V), rs1061170 (*CFH* Y402H & IAMDGC Locus 1.2, with rs570618 used as a proxy) and the *CFHR3/1* deletion tagging rs6677604. *P*-values (two-sided) for association with AMD were obtained through logistic regressions using the IAMDGC case/control cohort (13,378 controls, 17,541 cases) while controlling for age, sex and the first two genetic principal components. A *p*-value threshold of 5×10^{-8} (red line) was used. Only associations with variants with a frequency > 5% were tested. The significance of the association between AMD and rs1410996 (IAMDGC Locus 1.1) is lost when conditioning it to rs800292, rs1061170 and a *CFHR3/1* deletion tagging SNP. This is due to the fact that the IAMDGC Locus 1.1 does not perfectly tag the two independent common forms of genetic protection associated with the Chr1 AMD locus. Approximately 1.9% of controls and 1% of cases in the Utah & Iowa cohort and 1.6% of controls and 0.8% of cases in the IAMDGC cohort have a haplotype associated with protection against AMD that carries the protective A allele at rs800292 in the absence of the minor allele at IAMDGC Locus 1.1. The association between IAMDGC Locus 1.6 and AMD is also lost when conditioning it to rs800292, rs1061170 and the *CFHR3/1* deletion tagging SNP.

Supplementary Figure 3 Association between haplotypes based on rs187328863 (IAMDGC Locus 1.5), rs800292 (*CFH* I62V), rs1061170 (*CFH* Y402H & IAMDGC Locus 1.2, with rs570618 used as a proxy for the IAMDGC cohort), the *CFHR3/1* deletion-tagging rs12144939 (rs6677604 used as a proxy for the IAMDGC cohort) and rs61818925 (IAMDGC Locus 1.6), with associated effect sizes and *p*-values. Frequencies among Europeans from the 1000 Genomes Project phase 3 (1000 G EUR) are also provided. AMD odds ratios (OR), 95% confidence intervals (CI) and *p*-values (two-sided) were obtained using logistic regressions

including AMD case/control status as the dependent variable and age, sex and the first two genetic principal components for the IAMDGC cohort as covariates. Score statistics and *p*-values (two-sided) generated through chi-squared analyses are also shown. Bonferroni correction for multiple testing of 10 haplotypes = 0.005 (0.05/10).

Supplementary Figure 4 Heatmap of the log-likelihood of additive regression models conditioning variables present on the vertical axis to those on the horizontal axis. The log-likelihood for the model combining rs800292 (*CFH* I62V), the *CFHR3/1* deletion tagging rs6677604 and rs1061170 (*CFH* Y402H & IAMDGC Locus 1.2, with rs570618 used as a proxy) (boxed and highlighted) was used as a reference. This analysis was performed using logistic regressions with the IAMDGC case/control cohort (13,378 controls, 17,541 cases) while controlling for age, sex and the first two genetic principal components. A Bonferroni correction was applied to adjust for multiple testing. The addition of IAMDGC Locus 1.6 to regression models does not significantly decrease the log-likelihood.

Supplementary Figure 5 FHR-4 ELISA fit-for-purpose testing and validation. (a) The FHR-4 ELISA was run on one day to determine intra-assay variation and over three separate days to determine inter-assay variation in triplicates. Recombinant FHR-4A protein was spiked into reagent dilution buffer to determine sensitivity and precision or spiked into FH-depleted serum to determine dilutional linearity. Parallelism was determined using normal human serum at multiple dilutions. (b) To determine FHR-4 ELISA cross-reactivity, recombinant proteins (FH, FHL-1, FHR-1A, FHR-2, FHR-3, FHR-4A, FHR-4B and FHR-5) were spiked into FH-depleted serum at indicated concentrations. (c) To confirm FHR-4 specificity and selectivity in human plasma and serum samples, individuals with 0, 1 or 2 copies of the *CFHR1/4* genetic deletion were selected from the Utah & Iowa and Rapa Nui cohorts. Associations between plasma FHR-4/FHR-4A and CFHR4 or CFHR1/4 gene copy number were assessed using the the Mann-Whitney t-test. All *p*-values are two-sided.

Supplementary Figure 6 Variation of FHR-4 concentration with AMD status, AMD stage of severity and age. FHR-4 levels were log transformed and centered. The non-parametric Kruskal-Wallis test was used to test associations between FHR4-levels

and AMD status (a) and AMD severity (b). In all box plots the horizontal center lines correspond to the medians of the log-transformed FHR-4 distribution and the boxes delineate the 25th/75th percentile. The vertical solid lines represent the full range of the log-transformed FHR-4 distribution in each group. Dots beyond this line indicate potential outliers. In (c), linear regression of FHR-4 levels against age was used to determine the association between protein level and increasing age. All p-values are two-sided.

Supplementary Figure 7 Variation of FHR-4 concentration in plasma and in RPE/Bruch's membrane/choroid and vitreous lysates with genotype at rs1410996. FHR-4 levels were log transformed and centered. The Kruskal-Wallis test was used to test associations between FHR4-levels and genotype at rs1410996. Post-hoc pairwise comparisons were performed using the Conover-Iman test. All *p*-values are adjusted for multiple testing using the Bonferroni correction. In all box plots the horizontal center lines correspond to the medians of the log-transformed FHR-4 distribution and the boxes delineate the 25th/75th percentile. The vertical solid lines represent the full range of the log-transformed FHR-4 distribution in each group. Dots beyond this line indicate potential outliers.

Supplementary Figure 8 Variation of FHR-4 concentration in plasma with genotype at rs1410996, rs618189956, rs10494745 and rs7531555, by AMD status. FHR-4 levels were log transformed and centered. The non-parametric Kruskal-Wallis test was used to test associations between FHR4-levels and AMD status for each genotype. In all box plots the horizontal center lines correspond to the medians of the log-transformed FHR-4 distribution and the boxes delineate the 25th/75th percentile. The vertical solid lines represent the full range of the log-transformed FHR-4 distribution in each group. Dots beyond this line indicate potential outliers. None of the associations between FHR-4 levels and AMD were significant (two-sided $p > 0.05$).

Supplementary Figure 9 Haplotypes based on rs1410996, rs61818956, rs10494745 and rs7531555 among controls of the Utah & Iowa (1,587 subjects) and IAMDGC (13,378 participants) cohorts. Approximately 20% of chromosomes among these two groups carry both the FHR-4 reducing allele at rs1410996 (A) and rs7531555 (T). EAF: effect allele frequency.

Supplementary Figure 10 Comparison of Neutral haplotypes with and without the FHR-4 increasing allele (T) at rs61818956 and FHR-4 reducing allele (T) at rs7531555, along with a box plot showing variations in FHR-4 levels among subjects with Neutral/Neutral diplotypes stratified by genotype at these two QTLs. The Kruskal-Wallis test was used to test associations between log-transformed FHR4-levels and combinations of genotypes at rs7531555 and rs61818956. In all box plots the horizontal center lines correspond to the medians of the log-transformed FHR-4 distribution and the boxes delineate the 25th/75th percentile. The vertical solid lines represent the full range of the log-transformed FHR-4 distribution in each group. Dots beyond this line indicate potential outliers. All *p*-values are two-sided.

Supplementary Figure 11 Specificity of the monoclonal mouse anti-FHR-4 antibody (Cat. #MAB5980, R&D systems) and polyclonal sheep anti-FHR-4 antibody (Cat. #AF5980, R&D Systems, United States) to FHR-4A and FHR-4B and cross-reactivity with recombinants of the complement factor H family of proteins factor H (FH), factor H-like 1 (FHL-1), factor H related 1A (FHR-1A), factor H related 1B (FHR-1B), factor H related 2 (FHR-2), factor H related 3 (FHR-3) and factor H related 5 (FHR-5) by Western blot. The monoclonal mouse anti-FHR-4 antibody was used for immunohistochemistry and as the capture antibody for the custom sandwich ELISA. The polyclonal sheep anti-FHR-4 antibody was used as the detector antibody in the sandwich ELISA. Equimolar concentrations of recombinant protein were used. Both antibodies display a cross-reactivity with recombinant FHR-3.

Supplementary Figure 12 Negative control for FHR-4 immunohistochemistry.

Supplementary Note 1 Extended discussion of similarities and discrepancies of findings with those reported by Cipriani et al. (2020)[4], Cipriani et al. (2021)[5] and Lorés-Motta et al. (2021)[6]. See also Supplementary Table 8 for a detailed comparison with these studies.

Supplementary References.

Supplementary Table 1 Characteristics of the Utah & Iowa and IAMDGC case/control cohorts.

Demographic	Utah & Iowa Cohort			IAMDGC Cohort
	Utah	Iowa	Combined	
**N**	3,306	1,481	**4,787**	**30,919**
**Age, median (IQR)**	75.8 (13.2)	79.9 (11.1)	77.4 (12.7)	75 (13)(1)
**Males**	1,243	551	1,794	13,182
**Females**	2,057	930	2,987	17,737
**Controls**				
N	1,228	359	**1,587**	**13,378**
Age, median (IQR)	71.6 (10.5)	77.8 (12.6)	72.8 (11.6)	72 (13)(1)
Males	473	169	642	5,973
Females	755	190	945	7,405
**Cases**				
N	2,078	1,122	**3,200**	**17,541**
Age, median (IQR)	78.8 (12.1)	80.4 (10.3)	79.45 (11.6)	77 (11)(1)
Males	770	382	1,152	7,209
Females	1,302	740	2,042	10,332
Early AMD	445	245	690 (11.7%)	- (2)
Intermediate AMD	443	85	528 (10%)	5,132(3)
Late AMD (Atropy)	251	83	334 (10.4%)	2,606
Late AMD (Neovascular)	852	666	1,518 (47.4%)	8,331
Late AMD (Atrophy & Neovascular)	87	43	130 (4.1%)	1,472
Late AMD (Combined)	1,190	792	1,982 (61.9%)	12,409

(1) Does not include 624 subjects over 90, for which the exact age was not provided by the IAMDGC.
(2) Staging of AMD severity used by the IAMDGC did not include an Early AMD stage.
(3) Subjects with Intermediate AMD were excluded from the original GWAS performed by the IAMDGC.

Supplementary Table 2 Frequencies of Chr1 (*CFH-CFHR5*) and Chr10 (*ARMS2/HTRA1*) variants associated with AMD among individuals with European ancestry from the 1000 Genomes Project phase 3 (1000 G EUR), Utah & Iowa and IAMDGC controls and cases, with associated effect sizes and *p*-values. Odds ratios, 95% confidence intervals (CI) and *p*-values (two-sided) were determined using logistic regressions using AMD case/control status as the dependent variable and age, sex as well as for the first two genetic principal components for the IAMDGC cohort as covariates.

Variant, Label (Position) Major/Minor Allele	MAF 1000 G EUR	Utah & Iowa Cohort (1,587 controls, 3,200 cases)				IAMDGC (13,378 controls, 17,541cases)			
		MAF		OR (95% CI)	*p*-value*	MAF		OR (95% CI)	*p*-value**
		Controls	Cases			Controls	Cases		
**rs187328863**<br>**IAMGC Locus 1.5**<br>(chr1:196380158)<br>C/T (+)	0.028	0.029	0.053	2.12<br>[1.63; 2.74]	1.29e-08	0.017	0.034	2.30<br>[2.08; 2.55]	1.06e-57
**rs800292**<br>***CFH* I62V**<br>(chr1:196642233)<br>G/A (-)	0.260	0.241	0.142	0.52<br>[0.46; 0.58]	1.83e-29	0.237	0.138	0.51<br>[0.49; 0.53]	4.60e-202
**rs1061170**<br>***CFH* Y402H**<br>**IAMGC Locus 1.2**<br>(chr1:196659237)<br>T/C (+)	0.362	0.369	0.559	2.27<br>[2.06; 2.49]	1.16e-64	0.365[(1)]	0.554[(1)]	2.18[(1)]<br>[2.10; 2.26]	1.13e-422[(1)]
**rs1410996**<br>**IAMGC Locus 1.1**<br>(chr1:196696933)<br>G/A (-)	0.425	0.418	0.237	0.41<br>[0.37; 0.45]	1.04e-66	0.423	0.246	0.44[(2)]<br>[0.42; 0.46]	4.50e-415
**rs12144939**<br>***CFHR3/1* Deletion**<br>(chr1:196698945)<br>G/T (-)	0.190[(4)]	0.199	0.108	0.45<br>[0.39; 0.51]	1.43e-34	0.207[(2)]	0.116[(2)]	0.50[(2)]<br>[0.47; 0.52]	7.10e-187[(2)]
**rs61818925**<br>**IAMGC Locus 1.6**<br>(chr1:196815450)<br>G/T (-)	0.422	0.340	0.256	0.65<br>[0.59; 0.72]	1.68e-17	0.381	0.278	0.63<br>[0.60; 0.65]	2.11e-131

[(1)] Based on the perfect proxy rs570618 (chr1: 196657064) ($r^2 = 0.9914$, $D' = 1.0$) with major allele G and minor allele T.
[(2)] Based on rs6677604 (chr1:196686918), which is another tag for the *CFHR3/1* deletion with major allele G and minor allele A.
[*] Bonferroni correction for multiple testing of 6 variants = 0.008 (0.05/6).
[**] GWAS threshold for significance set at 5e-8.

Supplementary Table 3 Independent *cis*-expression quantitative trait loci (*cis*-eQTL) for *CFHR4* transcript levels. These variants were identified by performing a conditional association analysis using genotype and liver gene expression levels for 183 donors (129 males, 54 females) with European ancestry (median age 56 IQR 14.5) from the Genotype-Tissue Expression Project (GTEx) dataset, version 8[1]. Minor allele frequencies and linkage disequilibrium statistics were determined among these GTEx liver donors. Only common variants (frequency > 5%) were considered. Conditioned effect sizes and *p*-values (two-sided) were obtained using a backward stepwise linear regression model. Effect size and strength of association with AMD were determined by using the IAMDGC case/control cohort (13,378 controls, 17,541 cases) while controlling for age, sex and the first two genetic principal components.

**Variant (Position) Major/Minor Allele (Effect)**	**Rank**	**Conditioned effect size and *p*-value**	**MAF (GTEx)**	**Tagging SNP (Position) Major/Minor Allele (Effect) LD with Ranked Variant**	**Effect size and strength of association with AMD (IAMDGC Cohort)**
**rs559637118**<br>(chr1:196920148)<br>C/T (-)	1	$\beta = -0.55$<br>$p = 1.0 \times 10^{-08}$	0.445	**rs1410996**<br>**IAMDGC Locus 1.1**<br>(chr1:196696933)<br>G/A (-)<br>r^2= 1.0, *D'*= 1.0	OR = 0.44 [0.42; 0.45]<br>*p* = 4.5e-415
**rs1830959**<br>(chr1:196920148)<br>C/T (-)	2	$\beta = -0.48$<br>$p = 1.7 \times 10^{-05}$	0.254	**rs7531555**<br>(chr1:196929310)<br>C/T (-)<br>r^2= 1.0, *D'*= 1.0	OR = 0.58 [0.56; 0.61]<br>*p* = 2.7e-135

Supplementary Table 4 Independent *cis*-protein quantitative trait loci (*cis*-pQTL) identified for FHR-4. The study by Pietzner *et al.* (2021)[2] included 4,775 protein targets measured in plasma from 10,708 individuals with European ancestry (mean age 48.6 years, 53.3% women) from the Finland study. Association analyses were adjusted for age, sex, the first ten genetic principal components and test site. The study by (Gudjonsson *et al.* 2022)[3] included 4,782 protein targets measured in plasma collected from 5,368 individuals with European ancestry. Association analyses relied on linear regressions adjusted for age, sex, five genetic principal components and genotyping platforms. Effect size and strength of association with AMD were determined through logistic regression using the IAMDGC case/control cohort (13,378 controls, 17,541 cases) while controlling for age, sex and the first two genetic principal components. The minor allele for each variant, which correspond to the alternative allele used to determine odds ratios (OR), 95% confidence intervals and *p*-value for association with AMD, is underlined. All *p*-values are two-sided.

**Variant (Position) Effect/Non-Effect Allele (Effect)**	**Study**	**Unconditioned effect size estimate and *p*-value**	**Effect Allele Frequency (EAF)**	**Tagging SNP (Position) Effect/Non-Effect Allele (Effect) LD with Ranked Variant**	**Effect size and strength of association with AMD[(2)]**
**rs4915363**<br>(chr1:196905205)<br>T/C (+)	(Pietzner 2021)[2]	$\beta = 0.785$<br>$p = 1.3 \times 10^{-662}$	0.759	**rs7531555**<br>(chr1:196929310)<br>C/T (-)<br>r^2= 0.953, *D'*= 0.984	OR = 0.44 [0.42; 0.45]<br>*p* = 4.5e-415
**rs10494745**<br>(chr1:196887457)<br>A/G (-)	(Pietzner 2021)[2]	$\beta = -0.985$<br>$p = 8.0 \times 10^{-529}$	0.108	**rs10494745**<br>(chr1:196887457)<br>G/A (-)<br>-	OR = 1.68 [1.60; 1.77]<br>*p* = 7.8e-88
	(Gudjonsson 2022)[3]	$\beta = -0.899$<br>$p = 0.0$	0.165		
**rs61818956**<br>(chr1:196886223)<br>T/C (+)	(Pietzner 2021)[2]	$\beta = 0.877$<br>$p = 1.5 \times 10^{-652}$	0.180	**rs61818956**<br>(chr1:196886223)<br>T/C (+)<br>-	OR = 1.52 [1.45; 1.58]<br>*p* = 7.0e-82
**rs7413610**<br>(chr1:196855689)<br>A/G (+)	(Gudjonsson 2022)[3]	$\beta = -0.678$<br>$p = 1.7 \times 10^{-246}$	0.289	**rs7531555**<br>(chr1:196929310)<br>C/T (-)<br>r^2= 0.912, *D'*= 0.958	OR = 0.58 [0.56; 0.61]<br>*p* = 2.7e-135

Supplementary Table 5 Demographics of Utah & Iowa subjects among which FHR-4 plasma levels were measured, by Chr1 diplotype group and genotype at rs61818956, rs10494745 and rs7531555.

Demographic	All Participants	Risk/Risk	Neutral/Neutral	Prot-I62/Prot-I62	Prot-Del/Prot-Del
**N**	486	181	71	40	54
Females	288	113	41	23	35
**Age, median (IQR)**	75.0 (13)	76.0 (14.0)	75.0 (13.5)	75.0 (13.3)	74.0 (13.8)
**AMD Status**					
No AMD	235 (48.4%)	56 (30.9%)	28 (39.4%)	30 (75.0%)	32 (59.3%)
Early/Intermediate AMD	120 (24.7%)	59 (32.6%)	16 (22.5%)	4 (10.0%)	12 (22.2%)
Late AMD	131 (27.0%)	66 (36.5%)	27 (38.0%)	6 (15.0%)	10 (18.5%)
**rs61818956 (FHR-4 increasing allele: T)**					
CC	340	78	61	40	49
CT	112	85	6	0	0
TT	19	14	1	0	0
**rs10494745 (LFHR-4 reducing allele: A)**					
GG	359	90	68	37	49
AG	79	64	0	2	0
AA	14	14	0	0	0
**rs7531555 (FHR-4 reducing allele: T)**					
CC	316	163	49	1	45
CT	110	12	18	19	3
TT	41	1	1	20	0

Supplementary Table 6 Demographics of Utah & Iowa eye donors used in this study, by genotype at rs61818956, rs10494745 and rs7531555.

Demographic	All Donors (one eye only)	RPE/Bruch's membrane/ Choroid	Vitreous
**Donors**	229	199	147
Females	111	94	63
**Age, median (IQR)**	77 (13)	77 (13)	77 (12.5)
**rs61818956 (Effect Allele: T)**			
CC	166	145	112
CT	40	38	31
TT	8	5	4
**rs10494745 (Effect Allele: A)**			
GG	28	24	21
AG	17	15	11
AA	9	8	5
**rs7531555 (Effect Allele: T)**			
CC	135	117	92
CT	42	37	32
TT	33	30	20

Supplementary Table 7 Variants associated with systemic FHR-4 levels by GWAS and GWAS meta-analyses performed by Cipriani et al. (2020)[4], Cipriani et al. (2021)[5] and Lorés-Motta et al. (2021)[6] and independent sentinel *CFHR4* QTLs explaining these associations. The GWAS meta-analysis performed by Cipriani *et al*. (2020)[4] relied on 522 controls and 484 cases from the Cambridge & European Genetic Database (EUGENDA) cohorts. The GWAS on FHR-4 levels performed by Cipriani *et al*. (2021)[5] included 252 controls from the Cambridge cohort. The GWAS on FHR-4 levels carried out by Lorés-Motta *et al.* (2021)[6] relied on 202 controls from the EUGENDA cohort.

**Variant|Tagging SNP (Position) Major/Minor Allele (Effect on FHR-4 Levels)**	**Study**	**Independent Sentinel *CFHR4* QTLs (Position) Major/Minor Allele (Effect)**
**IAMGC Locus # 1.2**<br>**rs570618|rs1061170**<br>(chr1:196657064|chr1:196659237)<br>G/T|T/C (+)	(Cipriani 2020)[4]	**rs10494745**<br>(chr1:196887457)<br>G/A (-)<br>**rs61818956**<br>(chr1:196886223)<br>T/C (+)
	(Cipriani 2021)[5]	
	(Lorés-Motta 2021)[6]	
**IAMGC Locus # 1.1**<br>**rs10922109|rs1410996**<br>(chr1:196704632|chr1:196696933)<br>C/A|G/A (-)	(Cipriani 2020)[4]	**rs7531555**<br>(chr1:196961180)<br>C/T (-)
	(Cipriani 2021)[5]	
	(Lorés-Motta 2021)[6]	
**IAMGC Locus # 1.6**<br>**rs61818925**<br>(chr1:196815450)<br>G/T (-)	(Cipriani 2020)[4]	**rs7531555**<br>(chr1:196961180)<br>C/T (-)
	(Cipriani 2021)[5]	
	(Lorés-Motta 2021)[6]	
**rs56994749**<br>(chr1:196813692)<br>A/T (-)	(Lorés-Motta 2021)[6]	**rs7531555**<br>(chr1:196961180)<br>C/T (-)
**rs12047098**<br>(chr1:196835106)<br>T/C (-)	(Cipriani 2021)[5]	**rs7531555**<br>(chr1:196961180)<br>C/T (-)
**rs10494745**<br>(chr1:196887457)<br>G/A (-)	(Lorés-Motta 2021)[6]	**rs10494745**<br>(chr1:196887457)<br>A/G (-)
**rs4085749**<br>(chr1:196920148)<br>C/T (-)	(Cipriani 2021)[5]	**rs7531555**<br>(chr1:196961180)<br>C/T (-)

Supplementary Table 8 Comparison with studies from Cipriani et al. (2020)[4], Cipriani et al. (2021)[5] and Lorés-Motta et al. (2021)[6].

	Cipriani et al (2020)[4]	Cipriani et al (2021)[5]	Lores-Motta et al (2021)[6]	Zouache et al
***CFHR4* QTL Identification**				
**Type of QTL**	pQTL	pQTL	pQTL	*cis*-eQTL, pQTL
**Samples For Discovery**	522 controls<br>(Cambridge & Eugenda)	252 controls<br>(Cambridge)	202 controls<br>(Eugenda)	183 controls (GTEx)<br>10,708 controls (Pietzner et al 2021)[2]<br>5,368 controls (Gudjonsson et al 2022)[3]
**Variant Prioritization Method**	-	Instrumental Variable Analyses<br>GCTA- COJO	-	Forward-backward stepwise regression analysis (*cis*-eQTL)<br>GCTA (Pietzner et al 2021)[2]<br>GCTA-COJO (Gudjonsson et al 2022)[3]
**Refinement Methods**	-	-	Conditional Regression Analysis	Conditional Regression Analyses<br>Haplotype Analyses<br>Analyses of Levels among Genetic Combinations
**Samples For Validation and Refinements**	-	-	-	486 individuals<br>235 controls<br>120 early/intermediate AMD<br>131 late AMD
**AMD Association Analyses**				
**Variants/Haplotypes Considered**	IAMDGC 1.1, 1.2, 1.6 &<br>*CFHR3/1* deletion (common)<br>IAMDGC 1.3, 1.4, 1.5, 1.8 (rare)	IAMDGC 1.1, 1.2, 1.6 (common)<br>IAMDGC 1.3, 1.4, 1.5, 1.8 (rare)	IAMDGC 1.1, 1.2, 1.6 &<br>*CFHR3/1* deletion (common)<br>IAMDGC 1.3, 1.4, 1.5, 1.8 (rare)	IAMDGC 1.1, 1.2, 1.6, rs800292 & *CFHR3/1* deletion (common)
**Methods for Refinement of Associations**	-	-	-	Conditional Regression Analyses<br>Haplotype Analyses<br>Diplotype Analyses
**SNPs for Haplotypes**	IAMDGC 1.1, 1.2, 1.6 &<br>*CFHR3/1* deletion (common)<br>IAMDGC 1.3, 1.4, 1.5, 1.8 (rare)	-	IAMDGC 1.1, 1.2, 1.6 &<br>*CFHR3/1* deletion (common)<br>IAMDGC 1.3, 1.4, 1.5, 1.8 (rare)	IAMDGC 1.1, 1.2, 1.6, rs800292 & *CFHR3/1* deletion (common)

Supplementary Figure 1 Association between haplotypes based on rs800292 (*CFH* I62V), rs1410996 (IAMDGC Locus 1.1), rs1061170 (*CFH* Y402H, IAMDGC Locus 1.2) and the *CFHR3/1* deletion-tagging SNP rs12144939 (rs6677604 was used as a proxy for the IAMDGC cohort), with associated effect sizes and *p*-values. Frequencies among Europeans from the 1000 Genomes Project phase 3 (1000 G EUR) are also provided. AMD odds ratios (OR), 95% confidence intervals (CI) and *p*-values (two-sided) were obtained by using logistic regressions including AMD case/control status as the dependent variable and for age, sex and the first two genetic principal components for the IAMDGC cohort as covariates. Score statistics and *p*-values (two-sided) generated through chi-squared analyses are also shown. The IAMDGC Locus 1.1 imperfectly tags two independent protective haplotypes carrying the protective A allele at rs800292 or the deletion of *CFHR3/1*. On the protective haplotype H5, between 1.6% (IAMDGC cohort) and 1.9% (Utah & Iowa cohort) of controls do not carry the minor allele at the IAMDGC Locus 1.1 despite carrying the protective allele at rs800292. Bonferroni correction for multiple testing of 7 haplotypes = 0.0071 (0.05/7).

Haplotype (Effect)	rs800292	rs1061170	rs1410996	rs12144939	1000 G EUR Frequency	Utah & Iowa Cohort *1,587 controls 3,200 cases*						IAMDGC Cohort *13,378 controls 17,541 cases*					
						Frequency		Score Statistic	*p*-value*	OR (95 %CI)	*p*-value	Frequency		Score Statistic	*p*-value*	OR (95 %CI)	*p*-value
						Controls	Cases					Controls	Cases				
**H1** Risk	G	C	G	G	0.354	0.364	0.555	17.53	9.0e-69	1.58 [1.39; 1.80]	2.3e-14	0.362	0.552	45.34	0	1.57 [1.50; 1.65]	1.6e-81
**H2** Protective	A	T	A	G	0.242	0.210	0.125	-10.69	1.1e-26	0.60 [0.51; 0.70]	5.1e-12	0.209	0.124	-28.11	7.2e-174	0.61 [0.57; 0.64]	1.1e-64
**H3** Protective	G	T	A	T	0.210	0.192	0.104	-12.41	2.5e-35	0.51 [0.44; 0.61]	9.6e-17	0.197	0.111	-28.78	3.6e-182	0.58 [0.54; 0.61]	1.7e-75
**H4** Neutral	G	T	G	G	0.176	0.196	0.195	-0.77	0.44	**1.0 (reference)**	-	0.196	0.193	-2.27	0.02	**1.0 (reference)**	-
**H5** Protective	A	T	G	G	0.014	0.019	0.01	-3.34	8.5e-4	0.58 [0.37; 0.89]	1.1e-4	0.016	0.008	-9.09	9.5e-20	0.53 [0.45; 0.64]	8.7e-14
**H6** Protective	A	T	A	T	0.011	0.008	0.004	-3.52	4.3e-4	0.47 [0.24; 0.94]	9.0e-14	0.010	0.005	-8.57	1.0e-17	0.52 [0.42; 0.66]	1.2e-09
**H7** Neutral	G	T	A	G	0.011	0.007	0.004	-1.64	0.10	0.64 [0.33; 1.21]	0.3	0.007	0.006	-1.64	0.1	0.85 [0.68; 1.06]	0.1

* Based on the chi-squared test with 2 degrees of freedom.

Supplementary Figure 2 Manhattan plot for single-variant associations with AMD conditioned on rs800292 (*CFH* I62V), rs1061170 (*CFH* Y402H & IAMDGC Locus 1.2, with rs570618 used as a proxy) and the *CFHR3/1* deletion tagging rs6677604. *P*-values (two-sided) for association with AMD were obtained through logistic regressions using the IAMDGC case/control cohort (13,378 controls, 17,541 cases) while controlling for age, sex and the first two genetic principal components. A *p*-value threshold of 5×10^{-8} (red line) was used. Only associations with variants with a frequency > 5% were tested. The significance of the association between AMD and rs1410996 (IAMDGC Locus 1.1) is lost when conditioning it to rs800292, rs1061170 and a *CFHR3/1* deletion tagging SNP. This is due to the fact that the IAMDGC Locus 1.1 does not perfectly tag the two independent common forms of genetic protection associated with the Chr1 AMD locus. Approximately 1.9% of controls and 1% of cases in the Utah & Iowa cohort and 1.6% of controls and 0.8% of cases in the IAMDGC cohort have a haplotype associated with protection against AMD that carries the protective A allele at rs800292 in the absence of the minor allele at IAMDGC Locus 1.1. The association between IAMDGC Locus 1.6 and AMD is also lost when conditioning it to rs800292, rs1061170 and the *CFHR3/1* deletion tagging SNP.

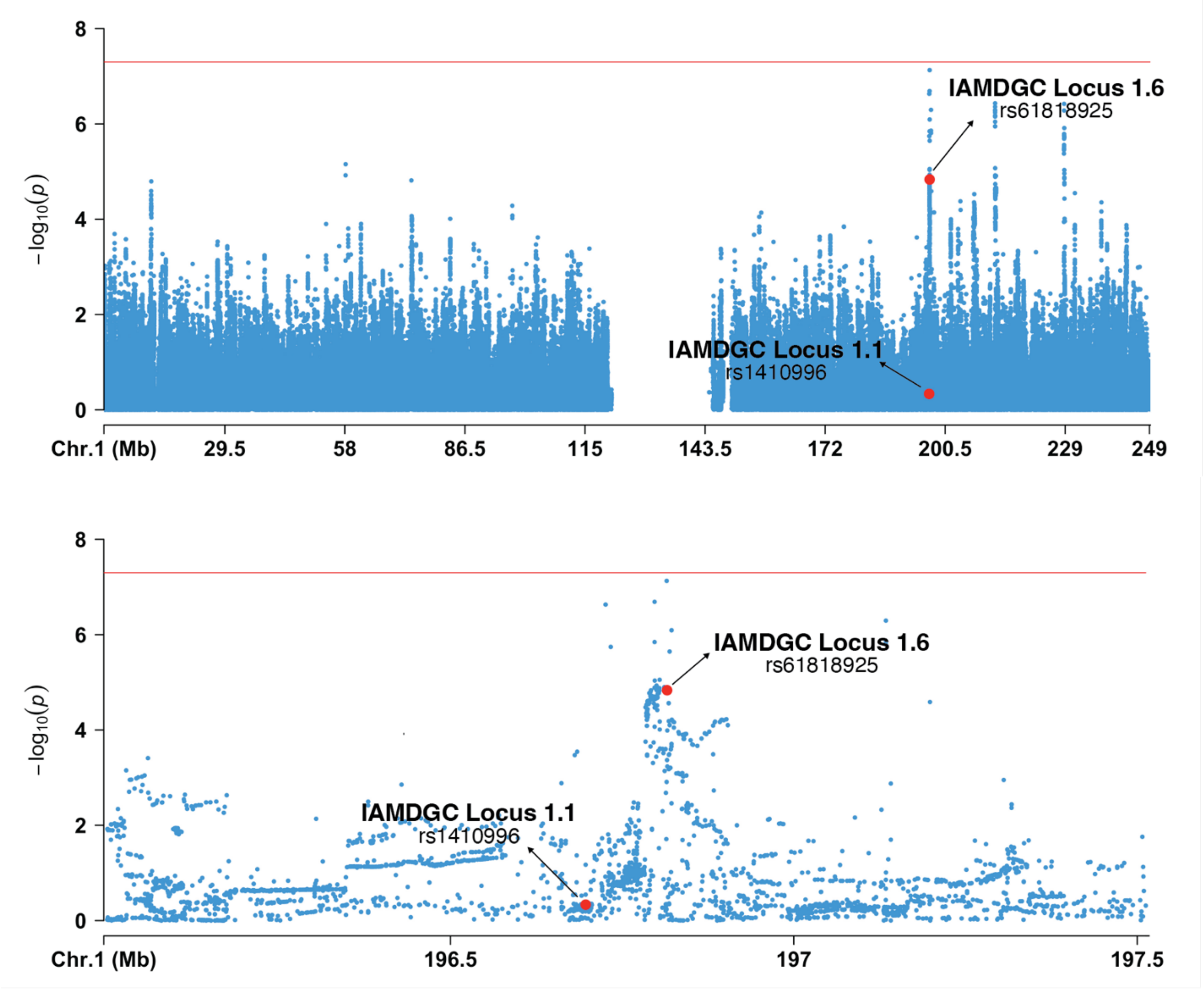

Associations for variants with frequency > 5% determined using the IAMDGC cohort (13,378 controls and 17,541 cases)

Supplementary Figure 3 Association between haplotypes based on rs187328863 (IAMDGC Locus 1.5), rs800292 (*CFH* I62V), rs1061170 (*CFH* Y402H & IAMDGC Locus 1.2, with rs570618 used as a proxy for the IAMDGC cohort), the *CFHR3/1* deletion-tagging rs12144939 (rs6677604 used as a proxy for the IAMDGC cohort) and rs61818925 (IAMDGC Locus 1.6), with associated effect sizes and *p*-values. Frequencies among Europeans from the 1000 Genomes Project phase 3 (1000 G EUR) are also provided. AMD odds ratios (OR), 95% confidence intervals (CI) and *p*-values (two-sided) were obtained using logistic regressions including AMD case/control status as the dependent variable and age, sex and the first two genetic principal components for the IAMDGC cohort as covariates. Score statistics and p-values (two-sided) generated through chi-squared analyses are also shown. Bonferroni correction for multiple testing of 10 haplotypes = 0.005 (0.05/10).

Haplotype (Effect)	rs187328863	rs800292	rs1060170	rs12144939	rs61818925	1000 G EUR Frequency	Utah & Iowa Cohort *1,587 controls* *3,200 cases*						IAMDGC Cohort *13,378 controls* *17,541 cases*					
							Frequency		Score Statistic	*p*-value*	OR (95 %CI)	*p*-value	Frequency		Score Statistic	*p*-value*	OR (95 %CI)	*p*-value
							Controls	Cases					Controls	Cases				
**H1** Risk	C	G	C	G	G	0.300	0.333	0.496	15.0	7.6e-51	1.56 [1.34; 1.83]	3.6e-18	0.340	0.511	41.55	0	1.53 [1.45; 1.62]	1.2e-51
**H2** Protective	C	A	T	G	T	0.223	0.207	0.123	-11.03	2.8e-28	0.60 [0.50; 0.72]	5.7e-11	0.219	0.128	-29.45	1.2e-190	0.60 [0.56; 0.64]	6.3e-57
**H3** Protective	C	G	T	T	G	0.153	0.190	0.102	-12.43	1.7e-35	0.52 [0.43; 0.63]	1.2e-12	0.176	0.094	-29.18	3.7e-187	0.54 [0.50; 0.58]	2.6e-71
**H4** Neutral	C	G	T	G	T	0.144	0.124	0.119	-1.08	0.3	**1.0 (reference)**	-	0.123	0.125	-1.06	0.30	**1.0 (reference)**	-
**H5** Neutral	C	G	T	G	G	0.066	0.080	0.080	-0.69	0.5	0.99 [0.79; 1.24]	0.8	0.080	0.074	-2.59	9.6e-03	0.97 [0.88; 1.06]	0.4
**H6** Protective	C	G	T	T	T	0.021	0.001[(1)]	0.002[(1)]	-[(2)]	-[(2)]	-[(2)]	-[(2)]	0.022	0.017	-8.08	6.4e-16	0.81 [0.68; 0.96]	1.3e-03
**H7** Risk	T	G	C	G	G	0.024	0.026	0.050	6.24	4.5e-10	2.22 [1.64; 3.0]	3.0e-11	0.017	0.035	13.72	7.7e-43	2.11 [1.86; 2.40]	7.8e-33
**H8** Protective	C	A	T	G	G	0.018	0.018	0.011	-2.61	8.8e-03	0.73 [0.47; 1.13]	1.6e-06	0.007	0.004	-7.10	1.2e-12	0.51 [0.38; 0.69]	6.7e-08
**H9** Protective	C	A	T	T	G	0.009	0.009	0.004	-4.10	4.1e-05	0.41 [0.20; 0.86]	4.6e-08	0.006	0.003	-8.01	1.1e-15	0.52 [0.36; 0.76]	1.2e-06
**H10** Risk	C	G	C	G	T	0.028	0.005	0.009	2.44	1.5e-02	2.03 [1.0; 4.12]	9.5e-07	0.005	0.006	2.60	9.3e-03	1.14 [0.86; 1.51]	0.3

(1) Frequency < 1% among the Utah residents from North and West Europe of the 1000 Genomes Project phase 3.
(2) Frequency too small to generate effect size.
* Based on the chi-squared test with 2 degrees of freedom.

Supplementary Figure 4 Heatmap of the log-likelihood of additive regression models conditioning variables present on the vertical axis to those on the horizontal axis. The log-likelihood for the model combining rs800292 (*CFH* I62V), the *CFHR3/1* deletion tagging rs6677604 and rs1061170 (*CFH* Y402H & IAMDGC Locus 1.2, with rs570618 used as a proxy) (boxed and highlighted) was used as a reference. This analysis was performed using logistic regressions with the IAMDGC case/control cohort (13,378 controls, 17,541 cases) while controlling for age, sex and the first two genetic principal components. A Bonferroni correction was applied to adjust *p*-values (two-sided) for multiple testing. The addition of IAMDGC Locus 1.6 to regression models does not significantly decrease the log-likelihood.

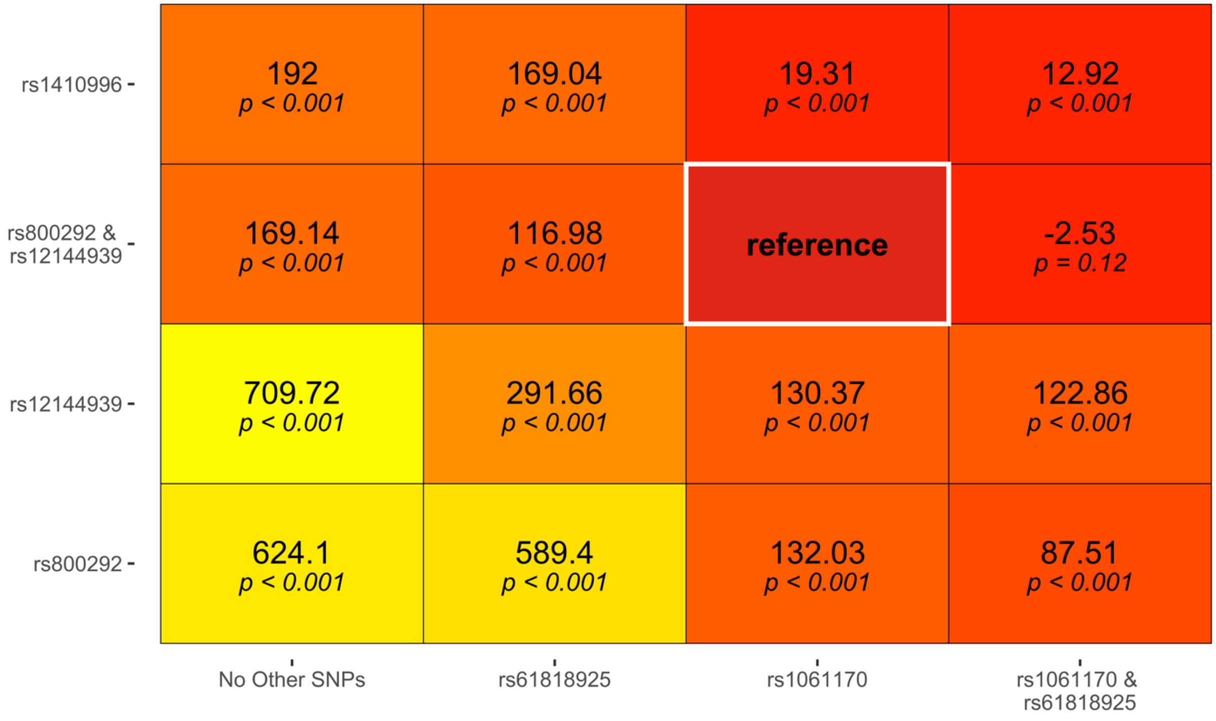

Supplementary Figure 5 FHR-4 ELISA fit-for-purpose testing and validation. (a) The FHR-4 ELISA was run on one day to determine intra-assay variation and over three separate days to determine inter-assay variation in triplicates. Recombinant FHR-4A protein was spiked into reagent dilution buffer to determine sensitivity and precision or spiked into FH-depleted serum to determine dilutional linearity. Parallelism was determined using normal human serum at multiple dilutions. (b) To determine FHR-4 ELISA cross-reactivity, recombinant proteins (FH, FHL-1, FHR-1A, FHR-2, FHR-3, FHR-4A, FHR-4B and FHR-5) were spiked into FH-depleted serum at indicated concentrations. (c) To confirm FHR-4 specificity and selectivity in human plasma and serum samples, individuals with 0, 1 or 2 copies of the *CFHR1/4* genetic deletion were selected from the Utah & Iowa and Rapa Nui cohorts. Associations between plasma FHR-4/FHR-4A and *CFHR4* or *CFHR1/4* gene copy number were assessed using the the Mann-Whitney t-test. All *p*-values are two-sided.

a

	Intra-Assay			Inter-Assay			Dilutional Linearity			Parallelism		
rFHR-4A Concentration (ng/mL) or Dilution	25	6.25	1.56	25	6.25	1.56	25	6.25	1.56	1:625	1:1250	1:2500
% Recovery	95.9	97.4	90.0	103.5	96.8	101.8	94.4	101.3	120.1	100	104.9	108.7
CV (%)	4.7	0.1	2.5	5.5	8.5	2.4	0.4	2.0	3.6	4.1	5.2	0.8

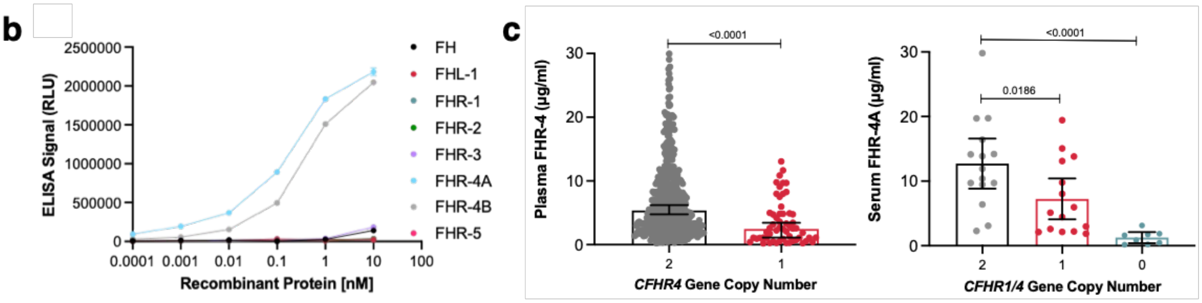

Supplementary Figure 6 Variation of FHR-4 concentration with AMD status, AMD stage of severity and age. FHR-4 levels were log transformed and centered. The non-parametric Kruskal-Wallis test was used to test associations between FHR4-levels and AMD status (a) and AMD severity (b). In all box plots the horizontal center lines correspond to the medians of the log-transformed FHR-4 distribution and the boxes delineate the 25th/75th percentile. The vertical solid lines represent the full range of the log-transformed FHR-4 distribution in each group. Dots beyond this line indicate potential outliers. In (c), linear regression of FHR-4 levels against age was used to determine the association between protein level and increasing age. All *p*-values are two-sided.

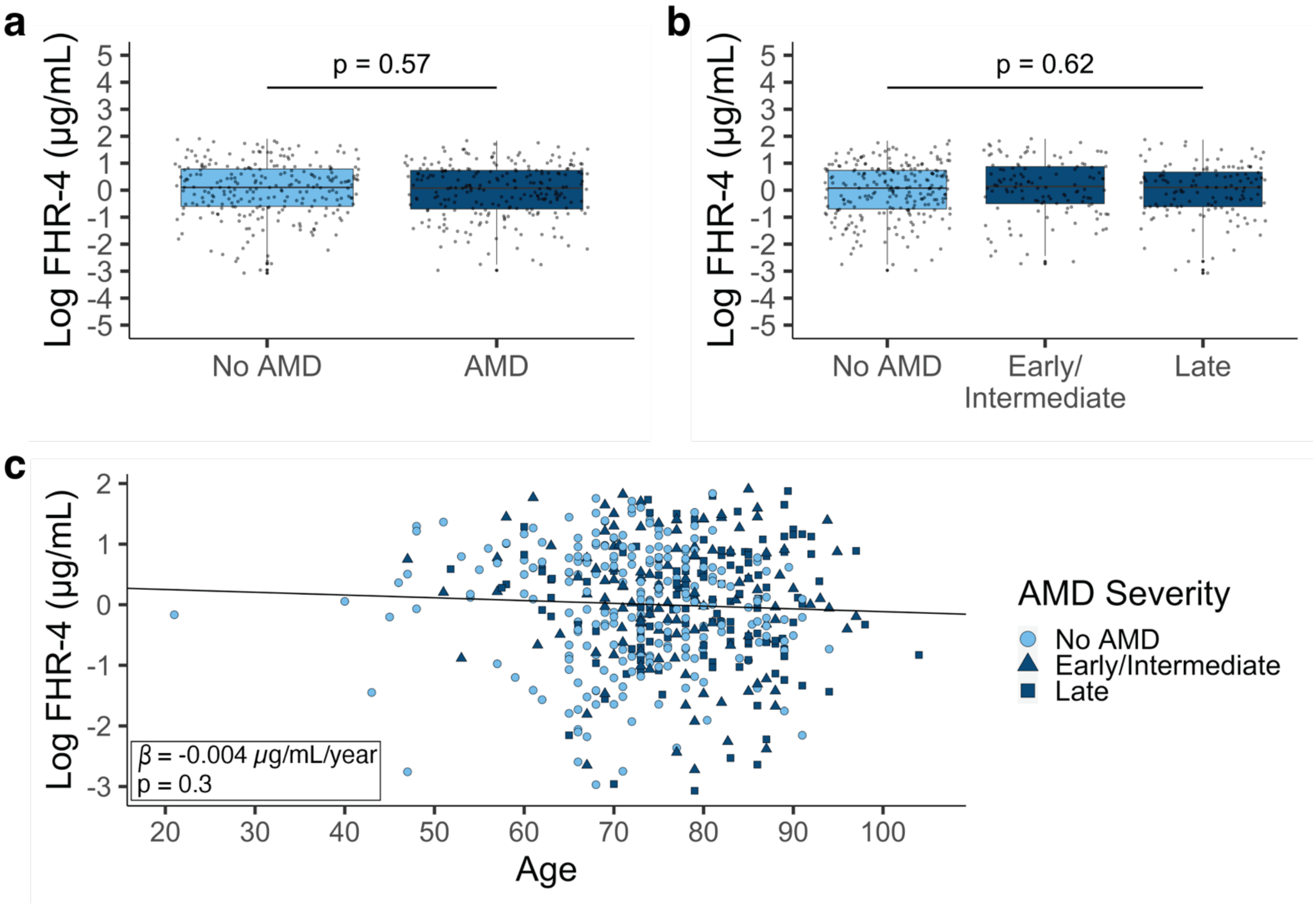

Supplementary Figure 7 Variation of FHR-4 concentration in plasma and in RPE/Bruch's membrane/choroid and vitreous lysates with genotype at rs1410996. FHR-4 levels were log transformed and centered. The Kruskal-Wallis test was used to test associations between FHR4-levels and genotype at rs1410996. Post-hoc pairwise comparisons were performed using the Conover-Iman test. All *p*-values are adjusted for multiple testing using the Bonferroni correction. In all box plots the horizontal center lines correspond to the medians of the log-transformed FHR-4 distribution and the boxes delineate the 25th/75th percentile. The vertical solid lines represent the full range of the log-transformed FHR-4 distribution in each group. Dots beyond this line indicate potential outliers.

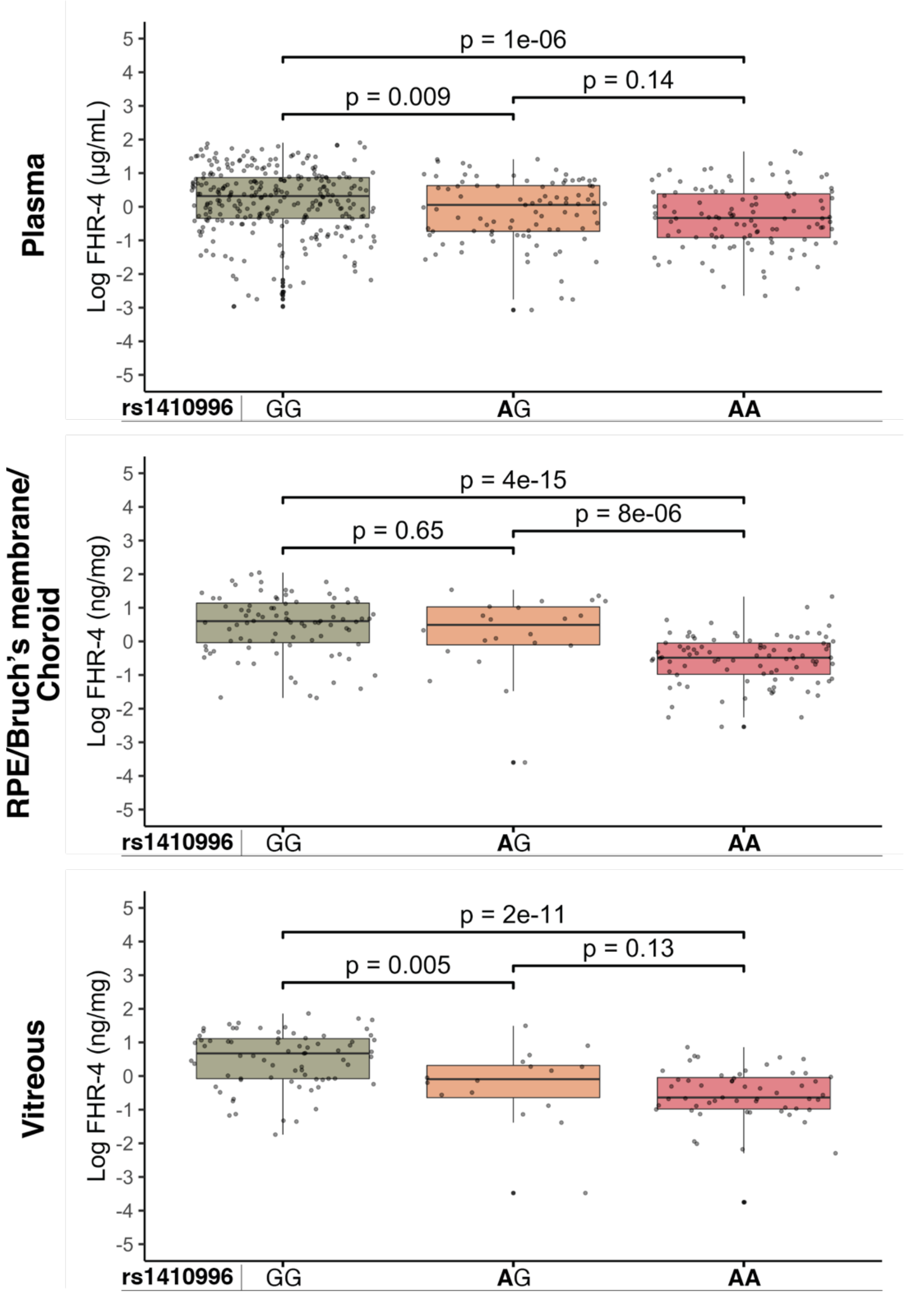

Supplementary Figure 8 Variation of FHR-4 concentration in plasma with genotype at rs1410996, rs618189956, rs10494745 and rs7531555, by AMD status. FHR-4 levels were log transformed and centered. The non-parametric Kruskal-Wallis test was used to test associations between FHR4-levels and AMD status for each genotype. In all box plots the horizontal center lines correspond to the medians of the log-transformed FHR-4 distribution and the boxes delineate the 25th/75th percentile. The vertical solid lines represent the full range of the log-transformed FHR-4 distribution in each group. Dots beyond this line indicate potential outliers. None of the associations between FHR-4 levels and AMD were significant (two-sided $p > 0.05$).

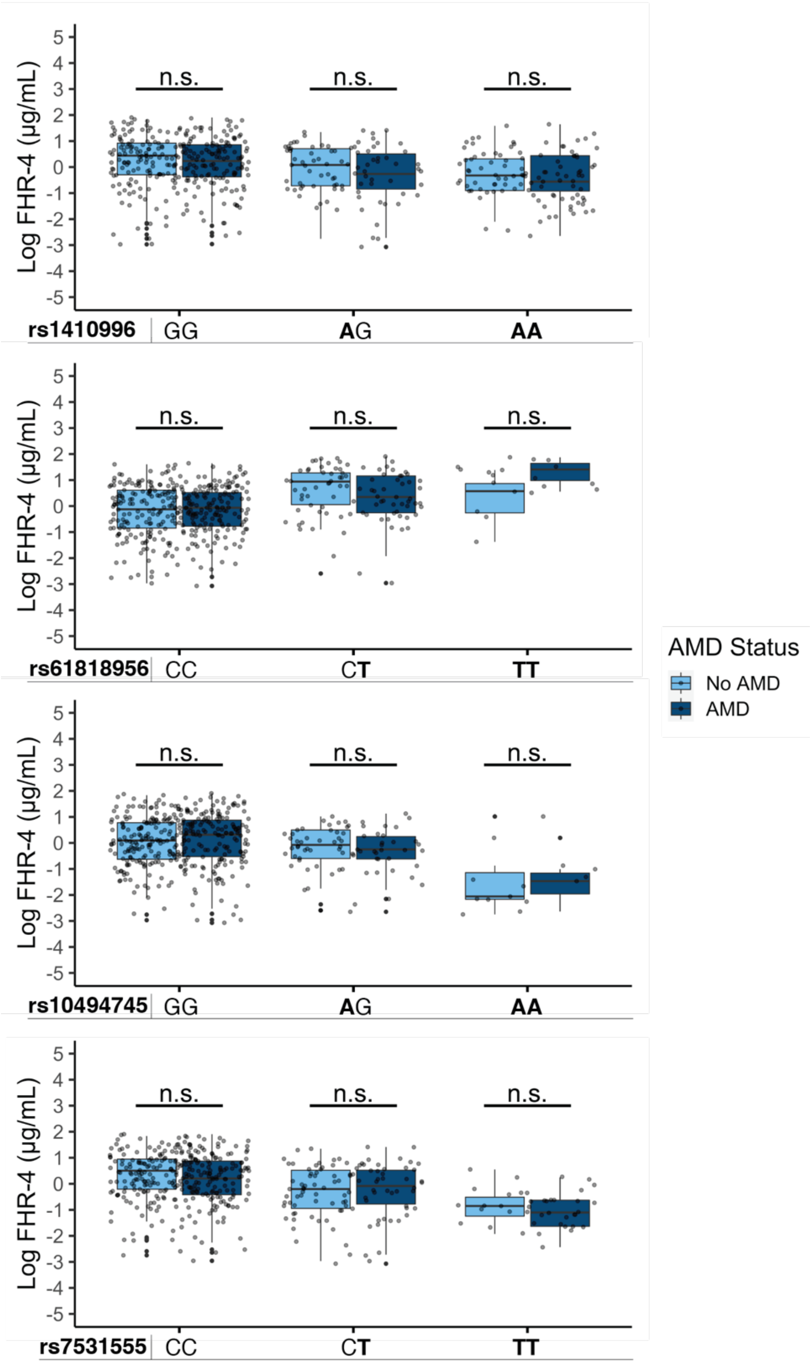

Supplementary Figure 9 Haplotypes based on rs1410996, rs61818956, rs10494745 and rs7531555 among controls of the Utah & Iowa (1,587 subjects) and IAMDGC (13,378 participants) cohorts. Approximately 20% of chromosomes among these two groups carry both the FHR-4 reducing allele at rs1410996 (A) and rs7531555 (T). EAF: effect allele frequency.

Utah & Iowa Cohort

(1,587 Controls)

Variant	EAF (Effect on FHR-4 Levels)	Haplotypes					
**rs1410996**	**A**: 0.418 (-)	G	A	A	G	G	G
**rs61818956**	**T**: 0.146 (+)	C	C	C	T	C	C
**rs10494745**	**A**: 0.103 (-)	G	G	G	G	A	G
**rs7531555**	**T**: 0.236 (-)	C	T	C	C	C	T
	**Haplotype Count**	1064	705	589	472	290	46
	**Haplotype Frequency**	0.319	0.217	0.196	0.145	0.098	0.023

IAMDGC Cohort

(13,378 Controls)

Variant	EAF (Effect on FHR-4 Levels)	Haplotypes					
**rs1410996**	**A**: 0.423 (-)	G	A	A	G	G	G
**rs61818956**	**T**: 0.169 (+)	C	C	C	T	C	C
**rs10494745**	**A**: 0.103 (-)	G	G	G	G	A	G
**rs7531555**	**T**: 0.239 (-)	C	T	C	C	C	T
	**Haplotype Count**	7771	5953	5312	4513	2720	423
	**Haplotype Frequency**	0.286	0.218	0.202	0.169	0.101	0.020

Supplementary Figure 10 Comparison of Neutral haplotypes with and without the FHR-4 increasing allele (T) at rs61818956 and FHR-4 reducing allele (T) at rs7531555, along with a box plot showing variations in FHR-4 levels among subjects with Neutral/Neutral diplotypes stratified by genotype at these two QTLs. The Kruskal-Wallis test was used to test associations between log-transformed FHR4-levels and combinations of genotypes at rs7531555 and rs61818956. In all box plots the horizontal center lines correspond to the medians of the log-transformed FHR-4 distribution and the boxes delineate the 25th/75th percentile. The vertical solid lines represent the full range of the log-transformed FHR-4 distribution in each group. Dots beyond this line indicate potential outliers. All *p*-values are two-sided.

Haplotype	rs800292	rs1061170	rs12144939	rs61818956	rs10494745	rs7531555	IAMDGC Cohort			Effect on FHR-4 Levels	AMD Effect
							OR	OR 95 %CI	*p*-value*		
**H9**	G	T	G	C	G	T	0.94	0.81 - 1.10	0.4	Reduced	Neutral
**H3**	G	T	G	C	G	C	reference	reference	-	Baseline	Neutral
**H7**	G	T	G	T	G	C	0.95	0.86 - 1.05	0.3	Elevated	Neutral

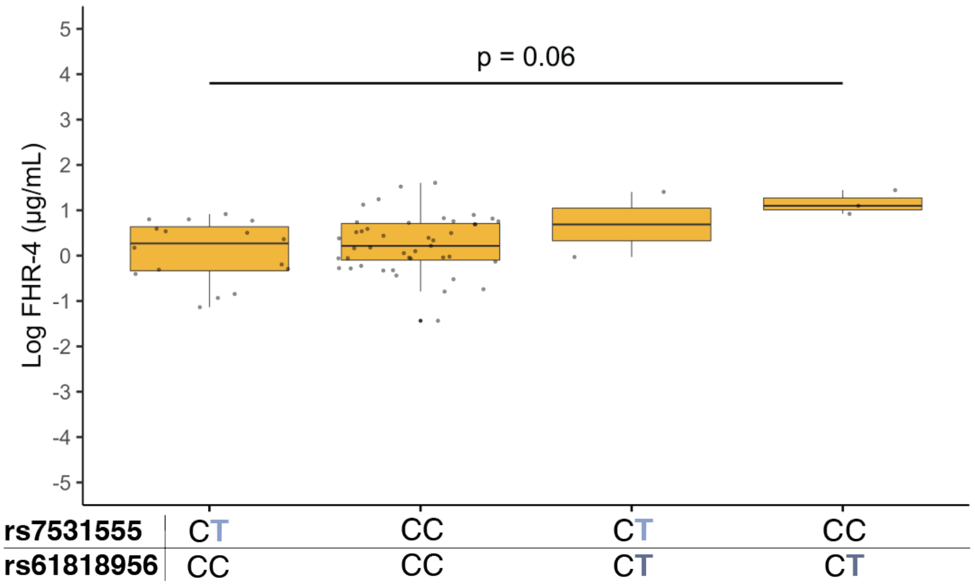

Supplementary Figure 11 Specificity of the monoclonal mouse anti-FHR-4 antibody (Cat. #MAB5980, R&D systems) and polyclonal sheep anti-FHR-4 antibody (Cat. #AF5980, R&D Systems, United States) to FHR-4A and FHR-4B and cross-reactivity with recombinants of the complement factor H family of proteins factor H (FH), factor H-like 1 (FHL-1), factor H related 1A (FHR-1A), factor H related 1B (FHR-1B), factor H related 2 (FHR-2), factor H related 3 (FHR-3) and factor H related 5 (FHR-5) by Western blot. The monoclonal mouse anti-FHR-4 antibody was used for immunohistochemistry and as the capture antibody for our custom sandwich ELISA. The polyclonal sheep anti-FHR-4 antibody was used as the detector antibody in the sandwich ELISA. Equimolar concentration of recombinant protein was used. Both antibodies display a cross-reactivity with recombinant FHR-3.

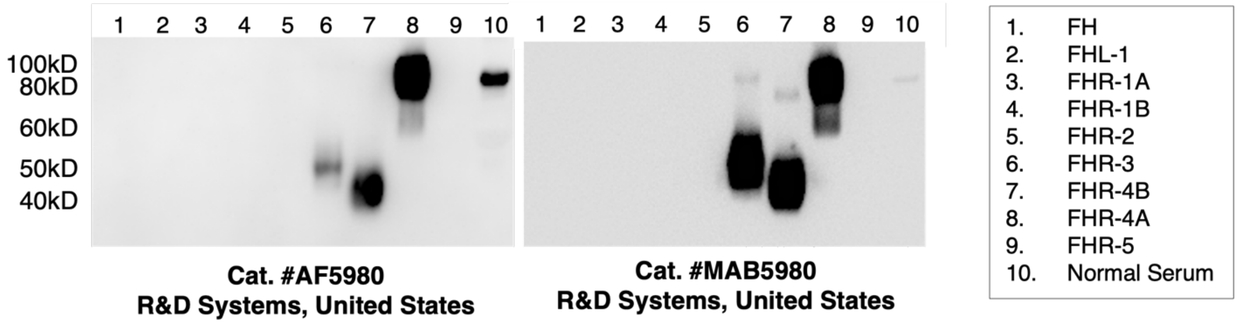

Supplementary Figure 12 Negative control for FHR-4 immunohistochemistry.

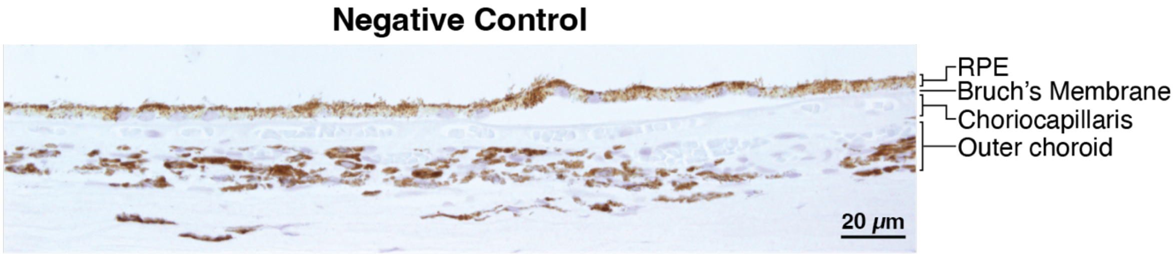

Supplementary Note 1 Extended discussion of similarities and discrepancies of findings with those reported by Cipriani et al. (2020)[4], Cipriani et al. (2021)[5] and Lorés-Motta et al. (2021)[6].
See also Supplementary Table 8 for a detailed comparison with these studies.

Discrepancy between our results and those of Cipriani et al (2020)[4] can be traced to three main points.

1. Cipriani et al (2020)[4] did not seek to identify causal *CFHR4* QTLs. Cipriani et al (2020)[4] identified *CFHR4* pQTLs by performing a GWAS on FHR-4 levels using 522 controls. They identified a total 543 variants with genome-wide significant association with FHR-4 levels in plasma. The variants that they chose from this list for further analyses were not selected based on refinement approaches designed to identify causal *CFHR4* pQTLs. They simply selected variants that had also been associated with AMD by the IAMDGC GWAS. We took a different approach in our study. We first sought to establish causal QTLs for FHR-4 independently of their association with AMD. We then dissected their association with AMD susceptibility. Cipriani et al (2020)[4] selected IAMDGC 1.1 (tagged by rs1410996 in our study) in their list of variants significantly associated with FHR-4 levels but did not seek to demonstrate that this variant was an independent QTL for *CFHR4*. In our study, we were able to confirm that IAMDGC 1.1 was a QTL for *CFHR4*. We then implemented refinement approaches based on conditional regression analyses, haplotype analyses and association analyses among groups with specific genetic combinations to show that the association between IAMDGC 1.1 and FHR-4 variation reported by Cipriani et al (2020)[4] was in fact entirely attributable to rs7531555.

2. Cipriani et al (2020)[4] did not seek to identify the most likely causal AMD variants. To demonstrate an overlap between AMD-associated variants and genetically driven variations in FHR-4, it is imperative to use AMD variants that are causal, or that are most likely causal variants. If the variants used are not causal, then any detected overlap may be due to linkage disequilibrium, making it potentially spurious. This is particularly relevant to 1q32 locus as it displays a high degree of linkage disequilibrium. In their study, Cipriani et al (2020)[4] simply used the index SNPs for 7 out of the 8 credible sets of variants independently associated with AMD by the IAMDGC GWAS in addition to a SNP tagging the *CFHR3/1* deletion. In our study, we sought to identify the most likely causal AMD variants through conditional genome-wide association analyses, haplotype analyses and diplotype analyses which relied on two large independent cohorts, including the one used by the IAMDGC for their GWAS. We showed that rs61818925 (IAMDGC 1.6) did not differentiate AMD susceptibility and was therefore not causal for AMD. We also demonstrated that IAMDGC 1.1 was not a causal AMD variant either (this is detailed in Supplementary Figure 1, 3 and 4 in addition to the first paragraph of the Results section). Its association with AMD stems from the fact that its minor allele overlaps with the protection-associated A allele at rs800292 (Prot-I62) and the *CFHR3/1* deletion haplotype. Having demonstrated this, we were able to robustly investigate possible overlaps between independent *CFHR4* QTLs and causal AMD-associated variants.

3. Cipriani et al (2020)[4] did not disentangle AMD variants from *CFHR4* QTLs. Rather, they used linkage disequilibrium to imply that variants responsible for variations in FHR-4 levels also affected AMD susceptibility without performing the necessary analyses to prove causality. Our manuscript sought to first establish causal AMD variants, then to identify independent QTLs for

FHR-4 levels. We were then able to dissect associations between causal AMD variants with variation in FHR-4, and between *CFHR4* QTLs and AMD susceptibility. In this way, we were able to overcome the challenge of LD and show that the variants affecting FHR-4 levels are not the variants that confer AMD risk.

Differences in FHR-4 levels between individuals with late AMD and controls has only been established in the combined Cambridge and EUGENDA cohorts used by Cipriani et al (2020)[4] and in later publications by Cipriani et al (2021)[5] and Lores-Motta et al (2021)[6]. Our study is the first to assess this difference in an independent cohort. We could not replicate this finding. Since we do not have access to the raw data from Cipriani et al (2020)[4], we can only make educated conjectures on why they observed that patients with late AMD have higher FHR-4 levels as compared to healthy controls. Because they have an opposite differential effect on FHR-4 levels and are present on AMD risk haplotypes, the frequencies of the minor alleles at rs61818956 and rs10494745 are likely to strongly influence the variability in FHR-4 levels among groups of individuals carrying high-risk haplotypes at the Chr1 AMD locus, which are more common among subjects with AMD. The presence of the level-reducing T at rs7531555 on protective haplotypes, which are more common among controls, is also likely to influence observed differences in levels between cases and controls.

It should however be noted that our study demonstrates that previously reported differences in FHR-4 levels between controls and patients with AMD is in fact inconsequential. Our rigorous analyses proves that genetic variations in FHR-4 levels are completely independent from AMD susceptibility. Many factors may explain why FHR-4 levels were observed to be higher among AMD patients; however, none of them influence AMD susceptibility in a significant way.

Of note, two GWAS conducted among 252 controls from the Cambridge cohort (Cipriani et al. 2021)[5] and 202 controls from the EUGENDA cohort (Lorés-Motta et al. 2021)[6], which were combined in the original publication from Cipriani et al. (2020)[4], reported findings that are consistent with ours (this analysis was added as Supplementary Table 7). The two studies relied either on objective variant prioritization technique (variants were selected independently from their potential involvement in AMD) or on conditional analyses for refinements. Lorés-Motta et al. (2021)[6] identified rs10494745 (not prioritized by Cipriani et al. 2020[4]) and a perfect proxy for rs1410996 (IAMDGC 1.1) as FHR-4 pQTLs (whose effect we show is attributable to rs7531555). The two variants reported by Cipriani et al. (2021)[5] were rs4085749, which is a perfect proxy for rs7531555 ($r^2 = 1.0$ and $D' = 1.0$), and rs12047098, which is in strong LD with rs7531555 ($r^2 = 0.826$ and $D' = 0.949$). None of these variants were prioritized in the original publication from Cipriani et al. (2020)[4]. The fact that neither study identified all independent *CFHR4* QTLs supports the idea of a bias due to small sample size in the three studies and demonstrates the importance of adequate variant prioritization techniques and refinements. It also shows the importance of using multiple datasets and genome-wide analysis methodologies for complete QTL discovery.

Supplementary References

1. GTEx Consortium. The GTEx Consortium atlas of genetic regulatory effects across human tissues. *Science* **369**, 1318–1330 (2020).
2. Pietzner, M. *et al.* Mapping the proteo-genomic convergence of human diseases. *Science* **374**, eabj1541 (2021).
3. Gudjonsson, A. *et al.* A genome-wide association study of serum proteins reveals shared loci with common diseases. *Nat. Commun.* **13**, 480 (2022).
4. Cipriani, V. *et al.* Increased circulating levels of Factor H-Related Protein 4 are strongly associated with age-related macular degeneration. *Nat. Commun.* **11**, 778 (2020).
5. Cipriani, V. *et al.* Beyond factor H: The impact of genetic-risk variants for age-related macular degeneration on circulating factor-H-like 1 and factor-H-related protein concentrations. *Am. J. Hum. Genet.* **108**, 1385–1400 (2021).
6. Lorés-Motta, L. *et al.* Common haplotypes at the CFH locus and low-frequency variants in CFHR2 and CFHR5 associate with systemic FHR concentrations and age-related macular degeneration. *Am. J. Hum. Genet.* **108**, 1367–1384 (2021).
