# Supplementary material for: Levels of complement factor H-related 4 protein do not influence susceptibility to age-related macular degeneration or its course of progression
Source: Nat Commun. 2024 Jan 10;15:443. doi: 10.1038/s41467-023-44605-0 (PMC10781981; doi:10.1038/s41467-023-44605-0)
Supplement: Supplementary file 3 — Reporting Summary [file 41467_2023_44605_MOESM3_ESM.pdf]

nature portfolio

Corresponding author(s): Moussa A. Zouache

Last updated by author(s): Dec 8, 2023

Reporting Summary

Nature Portfolio wishes to improve the reproducibility of the work that we publish. This form provides structure for consistency and transparency in reporting. For further information on Nature Portfolio policies, see our Editorial Policies and the Editorial Policy Checklist.

Statistics

For all statistical analyses, confirm that the following items are present in the figure legend, table legend, main text, or Methods section.

n/a	Confirmed	
☐	☒	The exact sample size (*n*) for each experimental group/condition, given as a discrete number and unit of measurement
☐	☒	A statement on whether measurements were taken from distinct samples or whether the same sample was measured repeatedly
☐	☒	The statistical test(s) used AND whether they are one- or two-sided<br>*Only common tests should be described solely by name; describe more complex techniques in the Methods section.*
☐	☒	A description of all covariates tested
☐	☒	A description of any assumptions or corrections, such as tests of normality and adjustment for multiple comparisons
☐	☒	A full description of the statistical parameters including central tendency (e.g. means) or other basic estimates (e.g. regression coefficient) AND variation (e.g. standard deviation) or associated estimates of uncertainty (e.g. confidence intervals)
☐	☒	For null hypothesis testing, the test statistic (e.g. *F*, *t*, *r*) with confidence intervals, effect sizes, degrees of freedom and *P* value noted<br>*Give P values as exact values whenever suitable.*
☒	☐	For Bayesian analysis, information on the choice of priors and Markov chain Monte Carlo settings
☐	☒	For hierarchical and complex designs, identification of the appropriate level for tests and full reporting of outcomes
☐	☒	Estimates of effect sizes (e.g. Cohen's *d*, Pearson's *r*), indicating how they were calculated

Our web collection on statistics for biologists contains articles on many of the points above.

Software and code

Policy information about availability of computer code

Data collection	All data pertaining to the Utah & Iowa cohorts, donors, or plasma proteome were collected and handled using commonly available software (Microsoft Excel).<br><br>The Genotype-Tissue Expression Project (GTEx) and IAMDGC datasets were downloaded from the database of Genotypes and Phenotypes (dbGAP) using the latest version of the SRA Toolkit (https://github.com/ncbi/sra-tools/wiki/).
Data analysis	R v4.2.0<br>Authors: Team RC<br>URL: https://www.r-project.org<br><br>pwr v1.2 (R package)<br>Authors: Stephane Champely, based on previous works by Claus Ekstrom and Peter Dalgaard, with contributions of Jeffrey Gill, Stephan Weibelzahl, Clay Ford, Aditya Anandkumar and Robert Volcic.<br>URL: https://github.com/heliosdrm/pwr<br><br>Haplo.stats v1.8.9 (R package)<br>Authors: Jason P. Sinnwell, DJ Schaid<br>URL: https://cran.r-project.org/web/packages/haplo.stats/index.html<br><br>Survival v3.3.1 (R package)<br>Authors: Terry M Therneau, Thomas Lumley, Atkinson Elizabeth, Crowson Cynthia

URL: https://cran.r-project.org/web/packages/survival/index.html

Survminer v0.4.9 (R package)
Authors: Alboukadel Kassambara, Marcin Kosinski, Przemyslaw Biecek, Scheipl Fabian
URL: https://cran.r-project.org/web/packages/survminer/index.html

Coxme v2.2-18.1 (R package)
Author: Terry M. Therneau
URL: https://cran.r-project.org/web/packages/coxme/index.html

Lmtest v0.9-40 (R package)
Authors: Torsten Hothorn, Achim Zeileis, Richard W. Farebrother, Clint Cummins, Giovanni Millo, David Mitchell
URL: https://cran.r-project.org/web/packages/lmtest/index.html

Conover.test v1.1.5 (R package)
Author: Alexis Dinno
URL: https://cran.r-project.org/web/packages/conover.test/index.html

PLINK v1.9
Authors: Shaun Purcell, Christopher Chang
URL: www.cog-genomics.org/plink/2.0/

PLINK v2.0
Authors: Shaun Purcell, Christopher Chang
URL: www.cog-genomics.org/plink/2.0/

QTLtools v.1.3.174
Authors: Olivier Delaneau, Halit Ongen, Manolis Dermitzakis
URL: https://qtltools.github.io/qtltools/

For manuscripts utilizing custom algorithms or software that are central to the research but not yet described in published literature, software must be made available to editors and reviewers. We strongly encourage code deposition in a community repository (e.g. GitHub). See the Nature Portfolio guidelines for submitting code & software for further information.

Data

Policy information about availability of data

All manuscripts must include a data availability statement. This statement should provide the following information, where applicable:

- Accession codes, unique identifiers, or web links for publicly available datasets
- A description of any restrictions on data availability
- For clinical datasets or third party data, please ensure that the statement adheres to our policy

Genotypes and gene expression data from The Genotype-Tissue Expression Project (GTEx) v8, associated demographic and tissue information (including age and ethnicity) and covariates used in eQTL analyses were obtained either through the database of Genotypes and Phenotypes (dbGAP) (accession number phs000424.v8.p2.c1, Project ID: 28906, PI: Moussa A. Zouache) [https://www.ncbi.nlm.nih.gov/projects/gap/cgi-bin/study.cgi?study_id=phs000424.v8.p2] or through the GTex dataset page (https://gtexportal.org/home/datasets).

Genotypes, case/control status and demographic information including age, gender and ethnicity from The IAMDGC GWAS were accessed through dbGaP (accession numbers phs001039.v1.c1 and phs001039.v1.c2, Project ID: 30476, PI: Moussa A. Zouache) [https://www.ncbi.nlm.nih.gov/projects/gap/cgi-bin/study.cgi?study_id=phs001039.v1.p1].

Genotypes among individuals with European ancestry from the 1000 Genomes Project phase 3 (1000 G EUR) were obtained from [https://www.ncbi.nlm.nih.gov/variation/tools/1000genomes/].

The list of pQTLs for CFHR4 published by Pietzner et al. (2021) (based on 10,708 subjects with European ancestry), which included for each variant the effect allele with associated frequency, an estimated effect size (adjusted for age, sex, the first ten genetic principal components and test site), p-value (stringent Bonferroni threshold for significance: $p < 1.004e-11$), and direction of association was downloaded from [https://omicscience.org/apps/pgwas/pgwas.download.php]. Outputs of the conditional analysis performed by the group were downloaded from the original publication ([https://www.science.org/doi/10.1126/science.abj1541], Supplementary Table S2).

The list of pQTLs for CFHR4 published by Gudjonsson et al. (2022) (based on 4,782 protein targets measured in serum from 5,368 individuals with European ancestry), which included for each variant effect allele and associated frequency, an estimated effect size (adjusted for age, sex, five genetic principal components and genotyping platforms) and p-value (Bonferroni threshold for significance: $p < 1.046e-11$) were obtained from the original publication [https://www.nature.com/articles/s41467-021-27850-z]. Outputs of the conditional analysis were obtained from the original publication (Supplementary Data 3).

All summary information pertaining to the Utah & Iowa cohorts or human donor eyes is included in the manuscript. Summary statistics of associations are also provided in the manuscript or its Supplementary Information. Raw data for FHR-4 levels in plasma and ocular tissue are provided in Source Data files. Clinical information and additional genetic data for the Utah & Iowa cohort are not available due to data privacy.

Research involving human participants, their data, or biological material

Policy information about studies with human participants or human data. See also policy information about sex, gender (identity/presentation), and sexual orientation and race, ethnicity and racism.

Reporting on sex and gender	Sex was considered in the study design; gender (shaped by social and cultural circumstances) was not. Sex was determined based on self-reporting. Disaggregated sex and gender data were not collected.<br><br>Effects associated with sex were accounted for in association analyses to adjust effect sizes and p-values for associations. Effects associated with sex were not assessed in isolation from other predictors or confounding factors, as this was not relevant to this particular study.
Reporting on race, ethnicity, or other socially relevant groupings	The population study was exclusively composed of individuals and donors of European ancestry.
Population characteristics	The population study was exclusively composed of individuals and donors of European ancestry aged 55 and above. Individuals and donors were selected on the basis of presence or absence of AMD in at least one eye, as independently determined by four investigators (co-authors).
Recruitment	Subjects from the Utah & Iowa cohort were recruited between 2009 and 2019 at the Sharon Eccles Steele Center for Translational Medicine (SCTM), John A. Moran Eye Center, University of Utah, United States and between 1999 and 2009 at the Cell and Molecular Biology Center, Department of Ophthalmology and Visual Sciences, University of Iowa, Iowa City, Iowa, United States as part of a case/control study of the genetic etiology of AMD. Participation in this study was voluntary and did not involve any monetary benefits. Association analyses relying on case/control status of participants from the Utah & Iowa cohort were not affected by self-selection biases. Analyses of the clinical course of progression of AMD were however affected by AMD severity at the time of recruitment, which differed between patients. We accounted for this potential bias by including AMD severity at the time of recruitment as a covariate in all survival analyses.<br><br>For the IAMDGC cohort, recruitment was performed in multiple centers in accordance with principles from the Declaration of Helsinki. All subjects provided informed consent, and protocols were reviewed and approved by the ethics committees of all participating centers/institutions. Association analyses relying on case/control status of participants from the IAMDGC cohort were not affected by self-selection biases.<br><br>Methods applied by the GTEx consortium for collection, preprocessing and selection of samples are detailed online at https://gtexportal.org/home/methods.<br><br>Methods applies by Pietzner et al. (2021) and Gudjonsson et al. (2022) to collect samples is detailed in their respective publications [https://www.science.org/doi/10.1126/science.abj1541] and [https://www.nature.com/articles/s41467-021-27850-z], respectively.
Ethics oversight	The study protocol was approved by the Institutional Review Boards of the University of Utah and University of Iowa. The list of organizations whose ethical committee approved the protocol may be found in the original publication [https://www.nature.com/articles/ng.3448], Supplementary Table 1.

Note that full information on the approval of the study protocol must also be provided in the manuscript.

Field-specific reporting

Please select the one below that is the best fit for your research. If you are not sure, read the appropriate sections before making your selection.

☒ Life sciences ☐ Behavioural & social sciences ☐ Ecological, evolutionary & environmental sciences

For a reference copy of the document with all sections, see nature.com/documents/nr-reporting-summary-flat.pdf

Life sciences study design

All studies must disclose on these points even when the disclosure is negative.

Sample size	All genetic association analyses (single variant/haplotype/diplotype) using the entirety of the Utah & Iowa cohorts were performed by including all eligible cases and controls and considering common variants (frequency > 5%) only. The robustness of all associations was validated independently against the GWAS performed by the IAMDGC, composed of 13,378 controls and 17,541 cases. Agreement between association analyses using the Utah & Iowa cohort and the IAMGC cohort was very strong.<br><br>All donors with European ancestry from the GTEx dataset were included for eQTL analyses. Sensitivity analyses were performed to determine the false discovery rate based on the available sample size.<br><br>Sample sizes for association analyses between FHR-4 levels and QTLs were initially determined on the basis of linear regression (as implemented by previous studies) by assuming that the proportion of variance explained by the three identified variants lied between 20 and 30%. Once generated, we observed that plasma and tissue FHR-4 levels were not in fact normally distributed, even after log transformation. We therefore resorted to non-parametric tests. Post-hoc power analyses indicated that the initially determined sample size provided a power

higher than 80% to detect associations between FHR-4 levels and QTLs in plasma and tissue. All power calculations were performed using the R package pwr v1.2 [https://github.com/heliosdrm/pwr] along the lines of Cohen, J. (1988). Statistical power analysis for the behavioral sciences (2nd ed.). Hillsdale,NJ: Lawrence Erlbaum.

Survival analyses included every AMD patient that met inclusion criteria.

Data exclusions

Only subjects and donors with European ancestry were retained. Only unrelated subjects and donors were retained. Subjects with poor genotype calls (<98.5%) were excluded. For genetic association analysis, subjects younger than 55 were excluded (this is required when looking into AMD associations since disease onset occurs later in life).

Replication

Analyses of associations were performed independently with the Utah & Iowa (1,587 controls, 3200 cases) and IAMDGC cohorts (13,378 controls, 17,541 cases). Agreement between these analyses was very strong.

Randomization

To allow for the refinement of associations between FHR-4 levels and AMD sought in this study, subjects and donor eyes were selected on the basis of the variants and haplotypes describing the spectrum of AMD susceptibility associated with the CFH-CFHR5 locus. Randomization of subjects and donors was therefore not relevant to this study.

Blinding

Blinding was not relevant to this study, as subjects and donor eyes had to be selected on the basis of the variants and haplotypes describing the spectrum of AMD susceptibility associated with the CFH-CFHR5 locus.

Reporting for specific materials, systems and methods

We require information from authors about some types of materials, experimental systems and methods used in many studies. Here, indicate whether each material, system or method listed is relevant to your study. If you are not sure if a list item applies to your research, read the appropriate section before selecting a response.

Materials & experimental systems

n/a	Involved in the study
☐	☒ Antibodies
☒	☐ Eukaryotic cell lines
☒	☐ Palaeontology and archaeology
☒	☐ Animals and other organisms
☒	☐ Clinical data
☒	☐ Dual use research of concern
☒	☐ Plants

Methods

n/a	Involved in the study
☒	☐ ChIP-seq
☒	☐ Flow cytometry
☒	☐ MRI-based neuroimaging

Antibodies

Antibodies used

Monoclonal mouse anti-FHR-4 antibody (Cat. #MAB5980, R&D systems, United States) (ELISA and Immunohistochemistry)

Polyclonal sheep anti-FHR-4 antibody (Cat. #AF5980, R&D Systems, United States) (ELISA)

Horse radish peroxidase-labeled polyclonal rabbit anti-sheep antibody (Cat. #313-035-045, Jackson ImmunoResearch, United States) (ELISA)

ImmPRESS-AP (alkaline phosphatase) horse anti-mouse IgG polymer detection kit (Cat. #MP-5402, Vector Laboratories, United States) (Immunohistochemistry)

Validation

Validation of the monoclonal mouse anti-FHR-4 antibody (Cat. #MAB5980, R&D systems, United States) for immunohistochemistry of human RPE/choroid was obtained through Western blot and negative controls (Supplementary Figures 11 and 12). We observed a cross-reactivity of both antibodies with recombinant FHR-3 on Western blot. Since equimolar concentrations of recombinant protein were used, the effect of this cross-reactivity on immunohistochemistry was negligible. This is reported and described in the manuscript.

Validation of the monoclonal mouse anti-FHR-4 antibody (Cat. #MAB5980, R&D systems, United States) and polyclonal sheep anti-FHR-4 antibody (Cat. #AF5980, R&D Systems, United States) for sandwich ELISA was performed through (1) western blot and (2) fit-for-purpose studies. Western blot showed cross-reactivity of both antibodies with recombinant FHR-3 but did not detect the protein in normal serum (Supplementary Figure 11). Since equimolar concentrations of recombinant proteins were used, the effect of this cross-reactivity is negligible and unlikely to have affected FHR-4 detection. FHR-4 sandwich ELISA cross-reactivity was assessed using recombinant his-tagged FH, FHL-1, FHR-1A and FHR-3 proteins purified in-house or purchased from commercial vendors: FHR-2 (Cat. #91-468, ProSci, United States), FHR-4A (Yurogen Inc, United States), FHR-4B (Cat. #5980-CH-050, R&D Systems, United States), FHR-5 (Cat. #3845-F5-050 R&D Systems, United States) and spiked into FH-depleted serum (1:1000 dilution) at equimolar concentrations ranging from 0.001 to 10 nM. To confirm FHR-4 specificity and selectivity in human plasma (Caucasian cohort) and serum (Rapa Nui cohort), subjects with 0, 1 or 2 copies of the CFHR1/4 genetic deletion were analyzed in the FHR-4 ELISA at 1:2500 dilution. These experiments confirmed that the combination of the monoclonal mouse anti-FHR-4 antibody (Cat. #MAB5980, R&D systems, United States) and polyclonal sheep anti-FHR-4 antibody (Cat. #AF5980, R&D Systems, United States) in a sandwich ELISA provided high specificity in detecting FHR-4.
